# Pharmacological Analysis of Ionotropic Glutamate Receptor Function in Neuronal Circuits of the Zebrafish Olfactory Bulb

**DOI:** 10.1371/journal.pone.0001416

**Published:** 2008-01-09

**Authors:** Rico Tabor, Rainer W. Friedrich

**Affiliations:** 1 Department of Biomedical Optics, Max-Planck-Institute for Medical Research, Heidelberg, Germany; 2 Friedrich-Miescher-Institute, Basel, Switzerland; University of Southern California, United States of America

## Abstract

Although synaptic functions of ionotropic glutamate receptors in the olfactory bulb have been studied in vitro, their roles in pattern processing in the intact system remain controversial. We therefore examined the functions of ionotropic glutamate receptors during odor processing in the intact olfactory bulb of zebrafish using pharmacological manipulations. Odor responses of mitral cells and interneurons were recorded by electrophysiology and 2-photon Ca^2+^ imaging. The combined blockade of AMPA/kainate and NMDA receptors abolished odor-evoked excitation of mitral cells. The blockade of AMPA/kainate receptors alone, in contrast, increased the mean response of mitral cells and decreased the mean response of interneurons. The blockade of NMDA receptors caused little or no change in the mean responses of mitral cells and interneurons. However, antagonists of both receptor types had diverse effects on the magnitude and time course of individual mitral cell and interneuron responses and, thus, changed spatio-temporal activity patterns across neuronal populations. Oscillatory synchronization was abolished or reduced by AMPA/kainate and NMDA receptor antagonists, respectively. These results indicate that (1) interneuron responses depend mainly on AMPA/kainate receptor input during an odor response, (2) interactions among mitral cells and interneurons regulate the total olfactory bulb output activity, (3) AMPA/kainate receptors participate in the synchronization of odor-dependent neuronal ensembles, and (4) ionotropic glutamate receptor-containing synaptic circuits shape odor-specific patterns of olfactory bulb output activity. These mechanisms are likely to be important for the processing of odor-encoding activity patterns in the olfactory bulb.

## Introduction

The first olfactory processing center in vertebrates, the olfactory bulb, transforms odor-specific patterns of sensory inputs across the array of glomeruli into spatio-temporal patterns of activity across the output neurons, the mitral cells. Processing of activity patterns in the olfactory bulb reduces the overlap between representations of related odors [1]–[3], rhythmically synchronizes odor-dependent ensembles of mitral cells [1], [4]–[6], and is likely to be important for additional computations involved in the analysis of an animal's molecular environment. The mechanistic basis of pattern processing in the olfactory bulb, however, is poorly understood.

**Figure 1:**
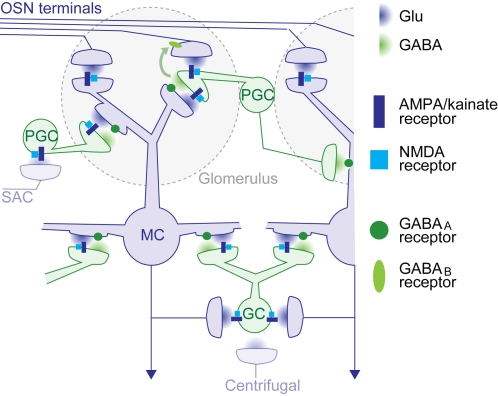
Simplified architecture of synaptic pathways in the olfactory bulb. Within glomeruli, glutamatergic olfactory sensory neurons provide excitatory synaptic input to mitral cells and a subpopulation of periglomerular cells via AMPA/kainate and NMDA receptors. Periglomerular cells also receive glutamatergic input from mitral cell dendrites and provide GABAergic output to mitral cells of the same and neighbouring glomeruli. In addition, GABA (green arrow) and dopamine (not shown) released from periglomerular cells reduces glutamate release from olfactory sensory neuron axon terminals by acting on GABA_B_ and D_2_ receptors, respectively, in the same glomerulus [23], [49]–[53]. In subglomerular layers, glutamate release from mitral cell dendrites and axon collaterals stimulates granule cells via AMPA/kainate and NMDA receptors. Granule cells release GABA back onto GABA_A_ receptors on the same and other mitral cells. Glutamate release from a mitral cell can therefore cause recurrent inhibition of the same mitral cell and lateral inhibition of other mitral cells via periglomerular and granule cells. These interactions, here collectively referred to as the mitral cell→interneuron→mitral cell pathway, can extend over distances corresponding to multiple glomeruli. An additional pathway mediating lateral inhibition that is not detailed in this scheme is the short axon cell (SAC)→periglomerular→mitral cell pathway identified in rodents [13], [54]. Centrifugal inputs from higher brain areas are also not shown in detail. Many of these inputs terminate on interneurons and are glutamatergic. Not included in the scheme are metabotropic glutamate receptors, interactions among interneurons in the granule cell layer [55], glutamate spillover [56], and a small glutamatergic subpopulation of granule cells [57]. Strong excitatory interactions across glomeruli, as revealed in the antennal lobe of Drosophila [58]–[60], have not been found in the vertebrate olfactory bulb. Abbreviations: OSN: olfactory sensory neuron, PGC: periglomerular cell, MC: mitral cell, GC: granule cell, SAC: short axon cell.

The synaptic architecture of neuronal circuits in the olfactory bulb is conserved across vertebrate classes [7], [8]. Within the sensory input modules of the olfactory bulb, the glomeruli, mitral cells can excite one another via gap junctions and fast volume transmission of glutamate [9]–[12]. Across glomeruli, synaptic interactions are mediated by interneurons, predominantly periglomerular and granule cells. Interactions among neurons associated with different glomeruli occur via various synaptic pathways that extend over multiple spatial scales and exert predominantly inhibitory effects on olfactory bulb output neurons [13], [14] (Fig. 1). The most prominent inter-glomerular synaptic pathway is the mitral cell→interneuron→mitral cell pathway, where periglomerular or granule cells are excited by glutamatergic mitral cell→interneuron synapses and feed back GABAergic inhibition onto the same and other mitral cells at interneuron→mitral cell synapses. This and other pathways (Fig. 1) shape spatio-temporal patterns of olfactory bulb output activity and may thereby optimize odor representations for processing in higher brain regions.

Experiments in brain slices have demonstrated that the activation of GABA release from interneurons can depend on NMDA receptor input [15], [16]. Glutamate release from mitral cells can cause long-lasting inhibitory GABA_A_ receptor currents in the same mitral cell even in the absence of action potential firing [15]–[17], partly by direct coupling of Ca^2+^ influx through the NMDA receptor to GABA release at the reciprocal dendro-dendritic synapse [18]–[20]. This mechanism is thought to mediate recurrent inhibition of the same presynaptic mitral cells because synaptic Ca^2+^ transients in granule cells are local events [21]. Strong inputs to interneurons trigger Na^+^ or Ca^2+^ action potentials that invade large portions of the dendritic tree and probably mediate inter-glomerular lateral inhibition among multiple mitral cells [21]–[23]. The relative strength of these different modes of inhibition during an odor response, however, is unclear.

Despite detailed insights into the molecular and biophysical properties of olfactory bulb neurons and synapses it remains unclear how synaptic interactions shape the spatio-temporal structure of olfactory bulb output activity in the intact circuit. To address this question, we took advantage of a preparation of the entire zebrafish brain that permits the combination of odor stimulation, electrophysiology, functional imaging and pharmacology. We concentrated on the role of ionotropic glutamate receptors, which comprise AMPA/kainate and NMDA receptors. Both receptor types are coexpressed at the olfactory sensory neuron→mitral cell synapse and at mitral cell→interneuron synapses [14], [24]. Hence, ionotropic glutamate receptors mediate most or all excitatory synaptic interactions among olfactory bulb neurons and are involved in multiple synaptic pathways (Fig. 1). While the combined blockade of AMPA/kainate and NMDA receptors abolished excitatory input to mitral cells, the selective blockade of each receptor type produced complex effects on the spatial and temporal patterning of olfactory bulb output activity. The results provide insights into the functions of synaptic circuits in the intact olfactory bulb, including the regulation of the total output activity, the mechanisms of interneuron activation, and the oscillatory synchronization of neuronal ensembles.

## Results

Mitral cells in the non-anesthetized olfactory bulb of zebrafish and other species exhibit pronounced fluctuations in membrane potential and fire spontaneous action potentials at irregular intervals. Odor stimulation causes stimulus- and mitral cell-dependent temporal modulations of firing frequency that can comprise successive excitatory and inhibitory epochs [2], [4], [25]–[28]. The population of mitral cells therefore responds to a given stimulus with a specific spatio-temporal pattern of activity. To analyze the role of glutamatergic synaptic interactions in this response, we pharmacologically manipulated AMPA/kainate and NMDA receptor function by bath-application of the selective antagonists 2,3-Dioxo-6-nitro-1,2,3,4-tetrahydropbenzo[f]quinoxaline-7-sulfonamide (NBQX; 5–10 µM) and D-(-)-2-Amino-5-phosphonopentanoic acid (AP5; 50–100 µM), respectively. We first measured the effect on odor responses of mitral cells in an explant preparation of the adult zebrafish brain by loose-patch extracellular and whole cell intracellular recordings. The panel of odor stimuli comprised two food extracts and 6 individual amino acids, which are natural odors for aquatic animals. The amino acid concentration used (10 µM) is in the intermediate physiological range and does not saturate glomerular odor responses in zebrafish [29].

### Combined blockade of AMPA and NMDA receptors

We first blocked all ionotropic glutamate receptors with NBQX and AP5. Spontaneous activity and odor responses were initially recorded for 15–20 min in the absence of drugs. Thereafter, NBQX and AP5 were washed in for 15 min and responses to the same stimuli were recorded again in the presence of the drugs. If recordings could be maintained for sufficient periods of time, drugs were washed out for at least 30 min and odor responses were measured again. The three recording conditions are designated “control”, “drug”, and “wash-out”, respectively.

**Figure 2:**
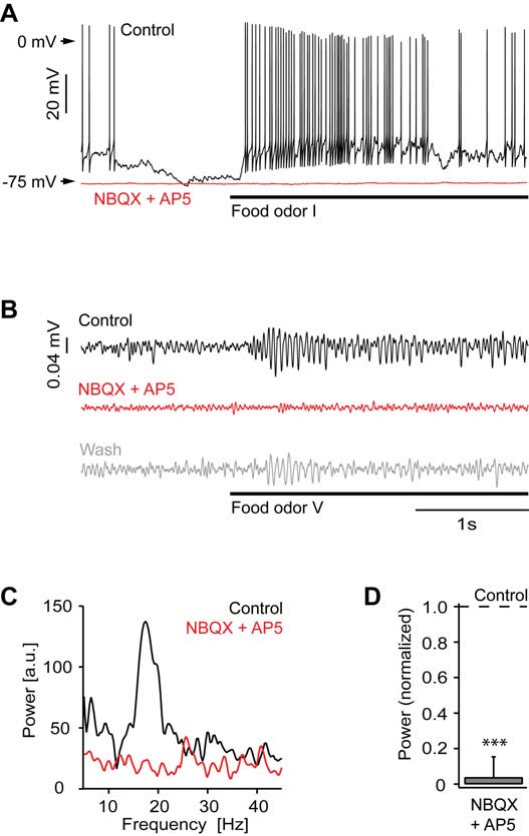
Ionotropic glutamate receptors are essential for odor responses of mitral cells. (A) Whole-cell recording from a mitral cell during odor stimulation (food extract; bar) before (black) and during (red) application of NBQX and AP5. (B) Local field potential recording during odor stimulation (food extract; bar) before (black) and during (red) application of NBQX and AP5 and after washout (gray). Traces are band-pass filtered between 8–43 Hz. (C) Power spectrum of local field potential traces (average of 6 trials; from unfiltered data) for the examples shown in (B). (D) Average local field potential power (15–30 Hz) in the presence of NBQX and AP5, normalized to control (n = 4 olfactory bulbs). ***, P<0.001.

In the presence of NBQX and AP5, spontaneous action potential firing was either completely abolished or became slow and periodic (n = 4 mitral cells; Fig. 2A). Subthreshold membrane potential fluctuations were reduced or eliminated and spontaneous fluctuations in the local field potential were decreased (Fig. 2A, B). Odor stimulation failed to elicit mitral cell depolarization and action potential firing (Fig. 2A). Similar results were obtained in all mitral cells, each of which was stimulated with 1–2 amino acid odors or food extracts that elicited a strong response under control conditions. Moreover, odor-evoked local field potential oscillations were completely abolished (Fig. 2B, C, D). In the presence of NBQX and AP5, the power in the 15–30 Hz band of the local field potential was significantly reduced to 4±1% of control (t-test: P<0.001). These results show that glutamatergic synaptic transmission is essential for responses of olfactory bulb neurons to odors, most likely because glutamate is the neurotransmitter of olfactory sensory neurons [30], [31].

### Blockade of AMPA/kainate receptors: effect on mitral cell responses

**Figure 3:**
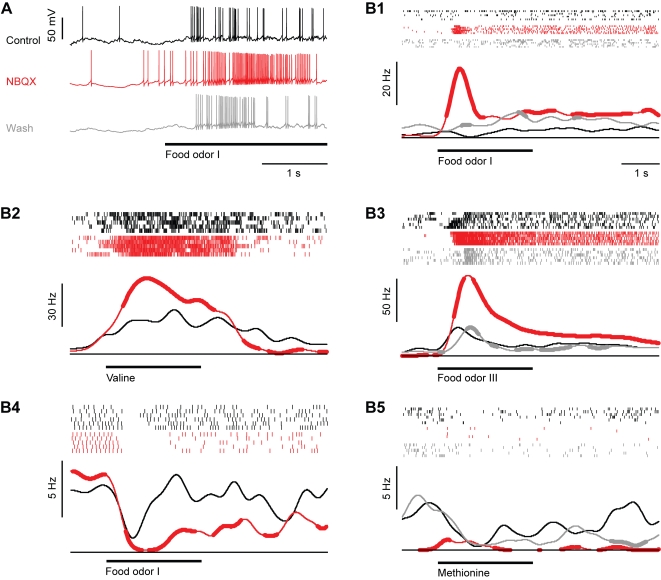
Effect of the AMPA/kainate receptor antagonist, NBQX, on odor responses of mitral cells. (A) Whole-cell recording of a mitral cell response to odor stimulation (food extract; bar) before (black) and during (red) bath-application of NBQX and after washout (gray). (B1–B5) Five examples illustrating effects of NBQX on odor responses. Ticks denote individual action potentials. Each row shows one trial. Black: control; red: during NBQX application; gray: after wash-out of NBQX. Continuous lines are peri-stimulus time histograms, averaged over all trials under each condition. Thick portions depict time bins where peri-stimulus time histograms were significantly different (Student's t-test; P<0.05) from the corresponding time bin in the control peri-stimulus time histogram (black). Bar indicates odor stimulation. Responses are from different cells and were recorded in the whole-cell, cell-attached or loose-patch configuration.

In contrast to the combined application of AMPA/kainate and NMDA receptor antagonists, the selective blockade of AMPA/kainate receptors by NBQX produced diverse effects on mitral cell activity. The spontaneous activity of individual mitral cells could decrease, remain similar, or even increase relative to control levels. On average, NBQX did not significantly change the spontaneous firing rate. Average spontaneous firing rates under control conditions and in the presence of NBQX were 6.6±3.61 Hz (mean±standard deviation) and 5.51±4.90 Hz, respectively (sign test: p = 0.23; n = 13 mitral cells). In most (5/6) mitral cells, steep subthreshold transients in the membrane potential were strongly reduced while slow fluctuations could still be observed (Fig. 3A).

All mitral cells were still odor-responsive in the presence of NBQX (n = 22 odor responses in 13 mitral cells; 1–3 different odors per mitral cell), but the magnitude and time course of odor responses was usually altered (Fig. 3A, B). Paradoxically, NBQX often enhanced transient periods of excitation shortly after response onset (Fig. 3B1–B3), while reductions in the amplitude of excitatory responses were rare. Inhibitory responses were prolonged in two cases (Fig. 3B4) and unchanged in one case. In two other cases, the sign of the response changed from an inhibition to a weak excitation (Fig. 3B5). In both of these cases, NBQX almost completely suppressed spontaneous activity. Changes in the sign of the response from excitatory to inhibitory were not observed. The effects of NBQX were at least partially reversed after wash-out.

**Figure 4:**
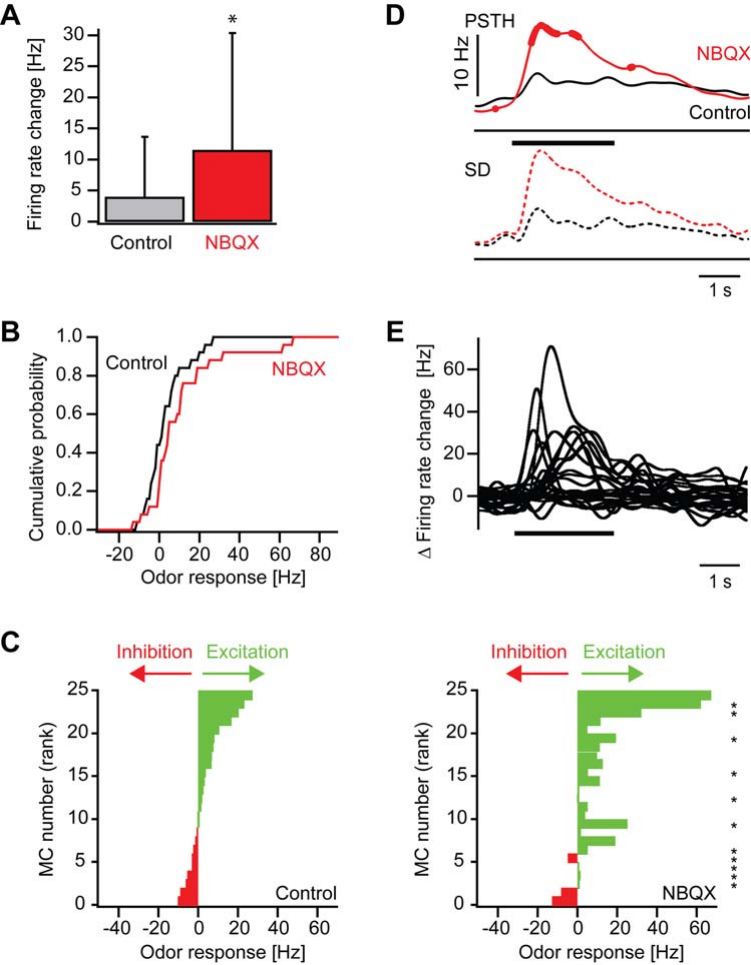
Effect of NBQX on odor responses of mitral cells: quantitative analysis. (A) Mean firing rate change evoked by odor stimulation before (control) and during NBQX treatment in the time window between 0.25 and 0.75 s after response onset. Error bars show standard deviation. *, P = 0.02 (sign test). (B) Cumulative distribution of odor-evoked firing rate changes in mitral cells before (control) and during NBQX application. (C) Left: mitral cell odor responses ranked according to the firing rate change measured before NBQX application. Right: Responses of the same mitral cells to the same odors in the presence of NBQX (same rank order as control). Asterisks denote responses that were significantly changed in the presence of NBQX (Student's t-test; P<0.05). (D) Top (continuous lines): average peri-stimulus time histogram of mitral cell odor responses before (control) and during NBQX treatment. Thick portions depict time bins where the peri-stimulus time histogram in the presence of NBQX was significantly different from the control peri-stimulus time histogram in the corresponding time bin (sign test; P<0.05). Dashed lines show standard deviation. (E) Differences of peri-stimulus time histograms (NBQX–control) for all mitral cell odor responses.

To quantify the effects of NBQX on odor responses we first compared the average odor-evoked firing rate change before and during NBQX treatment between 0.25 s and 0.75 s after response onset. On average, odor stimulation evoked an increase in mitral cell firing of 4.0±9.7 Hz above baseline under control conditions. This increase was significantly enhanced to 11.5±18.9 Hz by NBQX (sign test: P = 0.02; Fig. 4A). The cumulative distribution of response amplitudes was shifted to the right and saturated at higher frequencies (Fig. 4B), showing that smaller responses became less frequent and maximal response magnitudes were increased. However, not all responses were enhanced by NBQX and the effect of NBQX depended on the neuron and stimulus, suggesting that NBQX may also affect the pattern of activity across the population of mitral cells. We therefore compared responses of different mitral cells to different odors before and during application of NBQX in a diagram where responses are ranked according to their magnitude before drug application (Fig. 4C). Response patterns before and during application of NBQX showed obvious similarities, indicating that NBQX did not cause major changes in population activity patterns. Nevertheless, some, but not all, responses in the presence of NBQX were significantly different from control. Thus, the blockade of AMPA/kainate receptors not only scaled odor responses but also caused small changes in the distribution of activity across the mitral cell population.

To assess the effect of NBQX on the time course of mitral cell responses in more detail, we constructed peri-stimulus time histograms. The enhancement of the mean response of mitral cells was most pronounced during the early phase of the odor response (Fig. 4D). The effect of NBQX on individual responses was examined by subtracting the peri-stimulus time histogram measured before NBQX treatment from the corresponding histogram measured in the presence of NBQX (Fig. 4E). This analysis confirmed that NBQX increased odor responses in a subset of mitral cells, particularly during the initial phase of the odor response, while suppressive effects of NBQX were small.

### Blockade of NMDA receptors: effects on mitral cell responses

**Figure 5:**
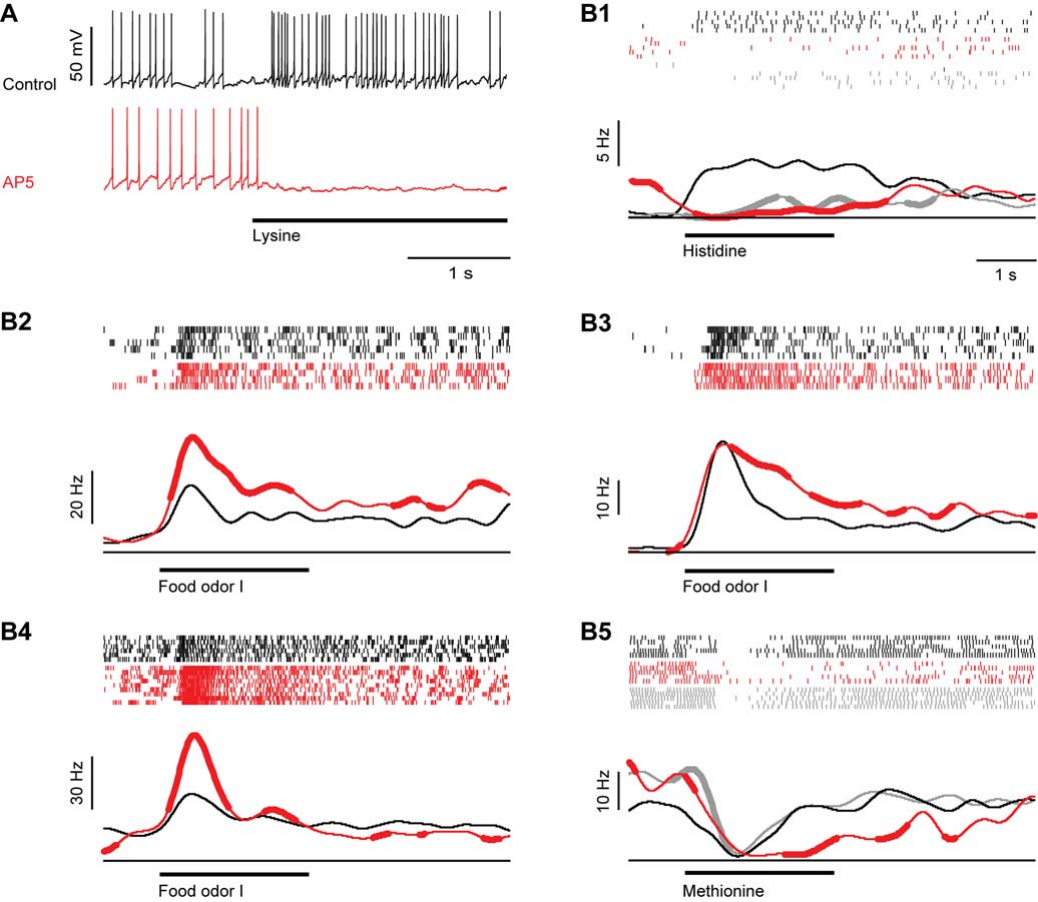
Effect of the NMDA receptor antagonist, AP5, on odor responses of mitral cells. (A) Whole-cell recording of a mitral cell response to odor stimulation (Lys, 10 µM; bar) before (black) and during (red) application of AP5. (B1–B5) Five examples illustrating effects of AP5 on odor responses. Conventions as in Fig. 3. Responses are from different cells and were recorded in the whole-cell, cell-attached or loose-patch configuration.

The selective blockade of NMDA receptors by AP5 had little effect on spontaneous mitral cell activity. In one mitral cell, spontaneous action potential firing was completely abolished, while it was slightly increased in others. The average spontaneous firing rates under control conditions and in the presence of AP5 were 8.6±6.5 Hz and 9.4±7.2 Hz, respectively (sign test: P = 1.00; n = 12 mitral cells). Fluctuations in the membrane potential in the presence of AP5 appeared largely unchanged as compared to control (Fig. 5A).

Odor responses were still observed after AP5 treatment in all recorded neurons (n = 25 responses from 12 mitral cells; 1–3 different odors per mitral cell), but the amplitude and time course were often changed (Fig. 5A, B). The effects caused by AP5 appeared more complex than those caused by NBQX. While excitatory response amplitudes were often slightly increased by AP5 (Fig. 5 B2–B4), decreases in response amplitude were also observed (Fig. 5A, 5B1). In some responses, AP5 affected mainly the initial transients (Fig. 5B4) whereas in other responses it also changed the later phases (Fig. 5B1, B2, B3, B5). Changes in the sign of the response amplitude were observed in 4 out of the 25 responses. In one case, a weak inhibitory response became excitatory while in the other three cases excitatory responses became inhibitory (Fig. 5A, B1). Effects of AP5 were at least partially reversible after washout.

**Figure 6:**
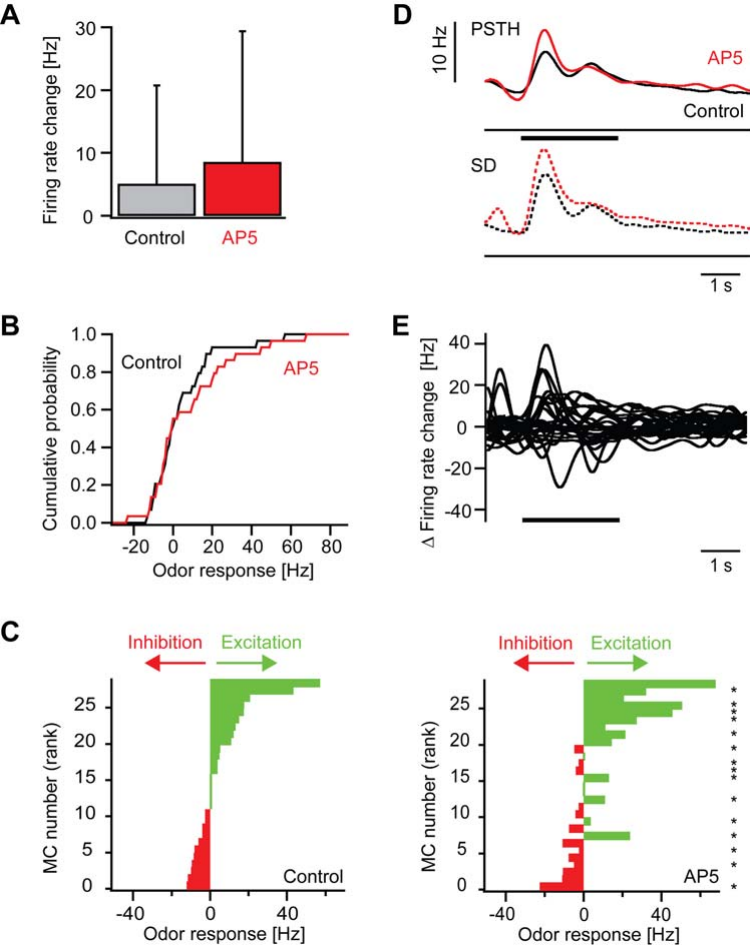
Effect of AP5 on odor responses of mitral cells: quantitative analysis. (A) Mean firing rate change evoked by odor stimulation before (control) and during AP5 treatment in the time window between 0.25 and 0.75 s after response onset. (B) Cumulative distribution of odor-evoked firing rate changes in mitral cells before (control) and during application of AP5. (C) Left: mitral cell odor responses ranked according to the firing rate change measured before application of AP5. Right: Responses of the same mitral cells to the same odors in the presence of AP5 (same rank order as control). Asterisks denote responses that were significantly changed in the presence of AP5 (Student's t-test; P<0.05). (D) Top (continuous lines): average peri-stimulus time histogram of mitral cell odor responses before (control) and during application of AP5. Thick portions depict time bins where the peri-stimulus time histogram in the presence of AP5 was significantly different from the control peri-stimulus time histogram in the corresponding time bin (sign test; P<0.05). Bottom (dashed lines): standard deviation. (E) Differences of peri-stimulus time histograms (AP5–control) for all mitral cell odor responses.

In the presence of AP5, the average firing rate change of mitral cells between 0.25 s and 0.75 s amounted to 8.5±20.8 Hz above baseline and was not significantly different from the average firing rate change of 5.0±15.7 Hz under control conditions (sign test: p = 0.71; Fig. 6A). The cumulative histogram of response amplitudes showed little or no change (Fig. 6B). The analysis of individual responses, however, revealed that AP5 increased some responses and decreased others. Consequently, the distribution of responses across the population of mitral cells in the presence of AP5 was different from control (Fig. 6C). Peri-stimulus time histograms revealed that the average time course of mitral cell firing was similar to control (Fig. 6D) but individual mitral cell responses could be increased or decreased, often within certain time windows (Fig. 6E). Largest changes were observed shortly after response onset, but later phases could also be affected. Hence, AP5 had little effect on the average magnitude and time course of the population response but caused complex changes of individual mitral cell responses and spatio-temporal activity patterns.

### Effects of ionotropic glutamate receptor antagonists on local field potential oscillations

**Figure 7:**
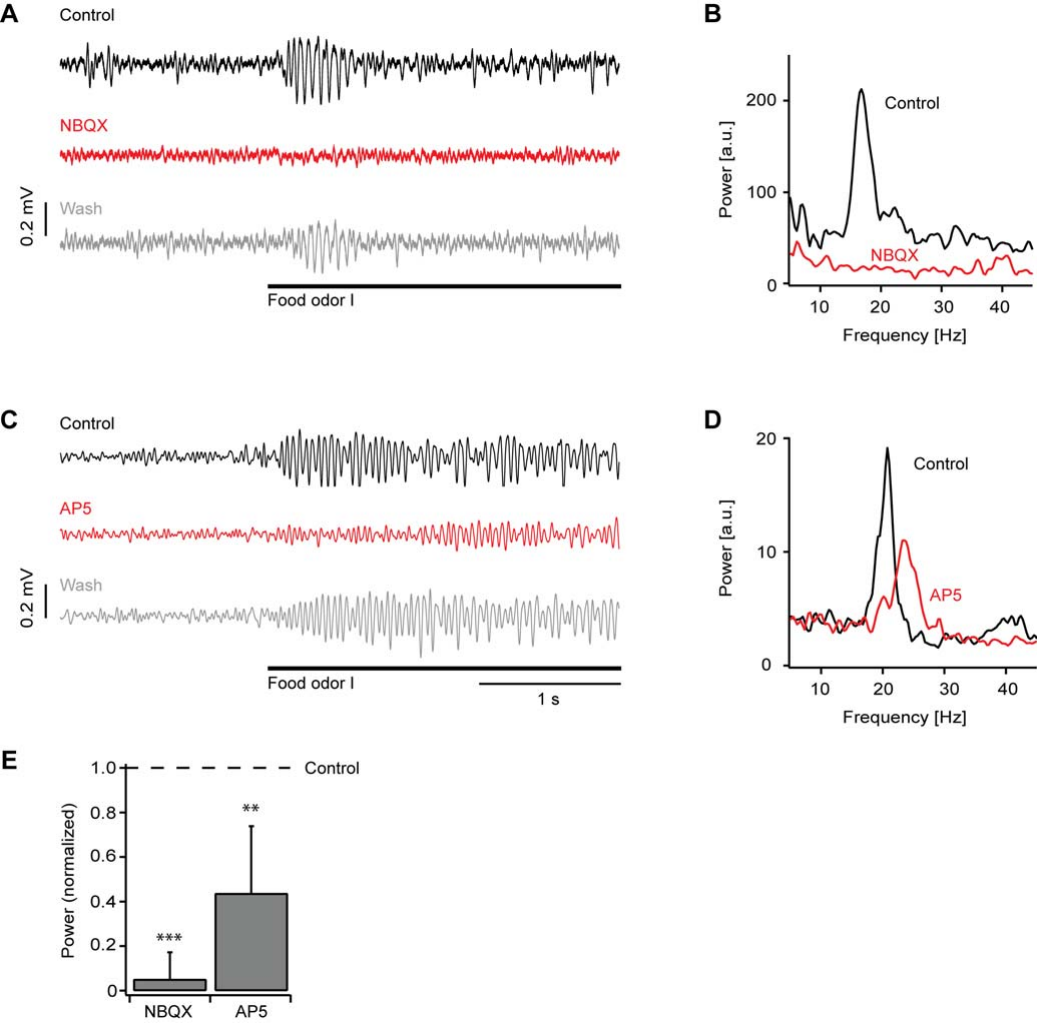
Effect of NBQX and AP5 on local field potential oscillations. (A) Example of a local field potential recording (bandpass-filtered 8–43 Hz) of an odor response (food odor; bar) before (black) and during application of NBQX (red) and after washout (gray). (B) Example of power spectra of local field potential responses before and during application of NBQX (average of 7 trials; calculated from unfiltered traces). (C) Example of a local field potential recording of an odor response before and during application of AP5 and after washout. (D) Example of power spectra before and during application of AP5 (average of 14 trials; calculated from unfiltered traces). (E) Average local field potential power (15–30 Hz) in the presence of NBQX (n = 6 olfactory bulbs) and AP5 (n = 4 olfactory bulbs), normalized to control. ***, P<0.001; **, P<0.01.

In the absence of drugs, all stimuli evoked local field potential oscillations with a frequency around 20 Hz (Fig. 7A–D). Because amplitudes were largest in response to food extracts, we concentrated on these stimuli for further experiments. NBQX completely abolished local field potential oscillations in response to food extracts (Fig. 7A, B, E), as observed in 6 olfactory bulbs. The power in the 15–30 Hz band was significantly reduced to 5±1% of control (t-test: P<0.001). AP5 reduced, but not completely abolished, local field potential oscillations (Fig. 7C–E), as observed in 4 olfactory bulbs. The power in the 15–30 Hz band was significantly reduced to 44±30% of control (t-test: P<0.01). Moreover, the oscillation frequency was slightly increased compared to control in all experiments (Fig. 7D). The effects of both drugs were reversible after washout (Fig. 7A, C).

### Measurements of odor-evoked activity patterns by 2-photon Ca^2+^ imaging

Although AMPA/kainate and NMDA receptors mediate excitatory synaptic input from olfactory sensory neurons to mitral cells, the blockade of one receptor type alone did not reduce the average excitation of mitral cells, suggesting that ionotropic glutamate receptors also influence mitral cell responses via other, multisynaptic pathways. We therefore analyzed the effect of ionotropic glutamate receptor antagonists on network activity patterns using 2-photon Ca^2+^ imaging after bolus loading of olfactory bulb neurons with the red-fluorescent Ca^2+^ indicator, rhod-2. Mitral cells and interneurons were distinguished by the expression of the mitral cell marker, HuC-YC [32], [33], that was detected simultaneously in a separate emission channel. Somatic Ca^2+^ signals reflect the spike output of individual mitral cells and interneurons [34] and are stable over hours [35]. 2-photon Ca^2+^ imaging therefore permits measurements of odor-evoked action potential firing from many neurons, including interneurons in deep layers that are difficult to record using electrophysiological methods.

**Figure 8:**
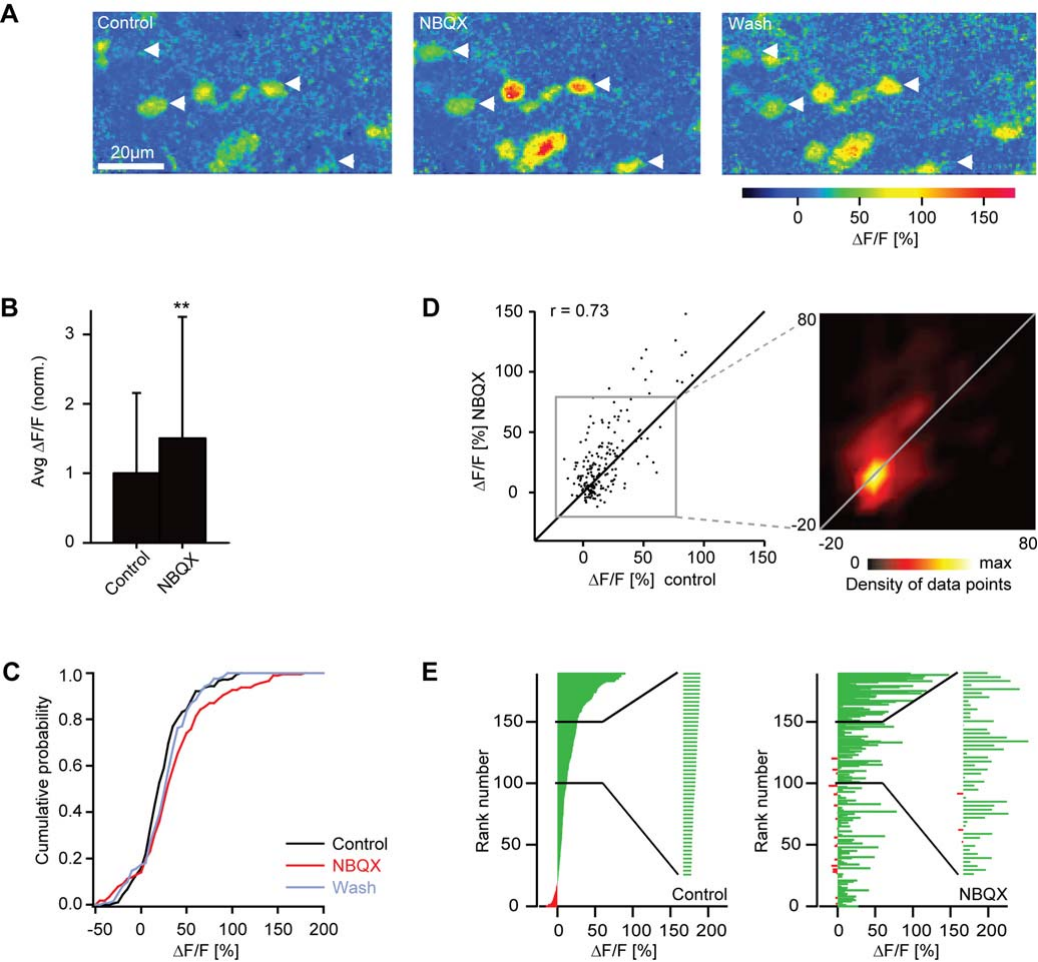
Effect of NBQX on mitral cell responses measured by 2-photon Ca^2+^ imaging. (A) Odor-evoked Ca^2+^ signals in mitral cells before, during and after application of NBQX (stimulus: Trp, 10 µM). Arrows depict somata of neurons identified as mitral cells by expression of the genetically encoded fluorescence marker HuC-YC. (B) Average somatic Ca^2+^ signals before (control) and during application of NBQX, normalized to control. Error bars show standard deviation. **, P = 0.002 (sign test). (C) Cumulative distribution of Ca^2+^ signal amplitudes before (black) and during application of NBQX (red) and after washout (gray). (D) Comparison of Ca^2+^ signal amplitudes evoked by the same odors in the same mitral cells before and during application of NBQX. Data were pooled over all cells, odors and animals (n = 190 responses). r, Pearson correlation coefficient. Inset shows the density of data points in the boxed region. Lines are diagonals with slope one. (E) Left: mitral cell odor responses ranked according to the Ca^2+^ signal before application of NBQX. Inset shows an enlargement of a subregion. Right: Responses of the same mitral cells to the same odors in the presence of NBQX, ranked in the same order as in the control.

We first examined the effect of glutamate receptor antagonists on odor-evoked Ca^2+^ signals of mitral cells using the same drug application protocol as before (Fig. 8A). In many mitral cells, NBQX increased the amplitude of odor-evoked Ca^2+^ signals, while decreases in response amplitude were rarely observed. On average, NBQX significantly increased Ca^2+^ signals to 150% of control (sign test: P = 0.002; Fig. 8B). Consequently, the cumulative distribution of response amplitudes was shifted towards higher amplitudes (Fig. 8C). At the level of individual mitral cells, the effect of NBQX varied in magnitude (Fig. 8D, E). The correlation between mitral cell activity patterns before and during NBQX treatment was 0.73 (Fig. 8D; n = 190 responses; pooled over all mitral cells and odors). As a control, we performed the same procedures in a different set of fish except that NBQX was omitted during the wash-in period. The correlation between activity patterns in these control experiments (r = 0.78; n = 126 responses) was slightly, but not significantly (P = 0.32), higher than in experiments using NBQX. Hence, NBQX increased the amplitude of the mitral cell population response but had little or no effect on the odor-evoked pattern of Ca^2+^ signals across the mitral cell population.

**Figure 9:**
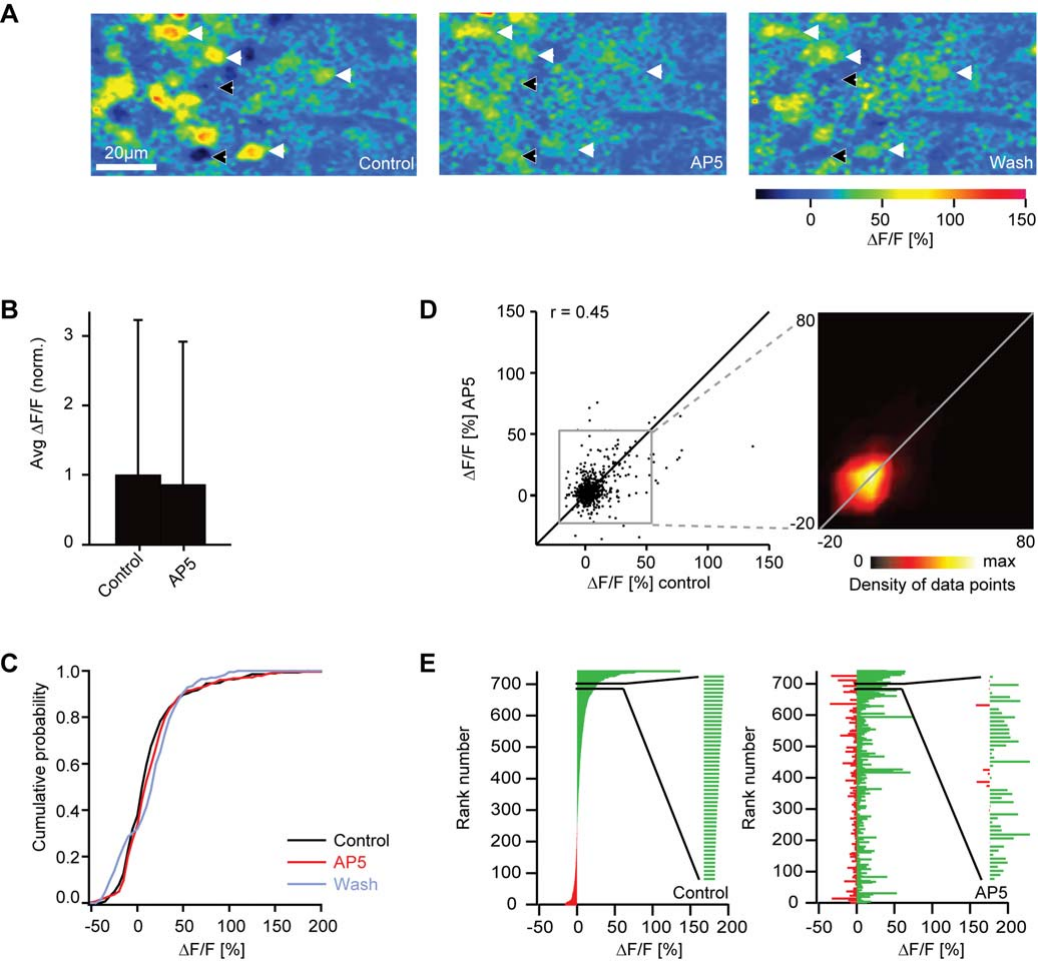
Effect of AP5 on mitral cell responses measured by 2-photon Ca^2+^ imaging. (A) Odor-evoked Ca^2+^ signals in mitral cells before, during and after application of AP5 (stimulus: food extract). Arrows depict somata of neurons identified as mitral cells by expression of the genetically encoded fluorescence marker HuC-YC. Black and white arrows show mitral cells whose response was increased and decreased, respectively, by AP5 treatment. (B) Average somatic Ca^2+^ signals before (control) and during application of AP5, normalized to control. Error bars show standard deviation. (C) Cumulative distribution of Ca^2+^ signal amplitudes before (black) and during application (red) of AP5 and after washout (gray). (D) Comparison of Ca^2+^ signal amplitudes evoked by the same odors in the same mitral cells before and during application of AP5. Data were pooled over all cells, odors and anminals (n = 742 responses). r, Pearson correlation coefficient. Inset shows the density of data points in the boxed region. Lines are diagonals with slope one. (E) Left: mitral cell odor responses ranked according to the Ca^2+^ signal before application of AP5. Inset shows an enlargement of a subregion. Right: Responses of the same mitral cells to the same odors in the presence of AP5, ranked in the same order as in the control.

Blockade of NMDA receptors by AP5 had diverse effects on odor-evoked Ca^2+^ signals of individual mitral cells, including increases and decreases of the response (Fig. 9A). The average response amplitude was 86% of control and not significantly different from control (sign test: P = 0.26; Fig. 9B). The cumulative histogram of response amplitudes showed no obvious change (Fig. 9C). However, the activity pattern across the mitral cell population differed from control because some responses were increased while others were decreased (Fig. 9D, E). The correlation between activity patterns before and during AP5 treatment was 0.45 (n = 742 responses) and significantly different from control (r = 0.78; n = 126 responses; P<0.001). Hence, AP5 did not significantly affect the mean response amplitude of mitral cells but changed the pattern of activity across the population. The effects of AP5 and NBQX on odor-evoked patterns of Ca^2+^ signals across mitral cells are therefore consistent with those observed in electrophysiological experiments.

### Effects of ionotropic glutamate receptor antagonists on interneuron activity

**Figure 10:**
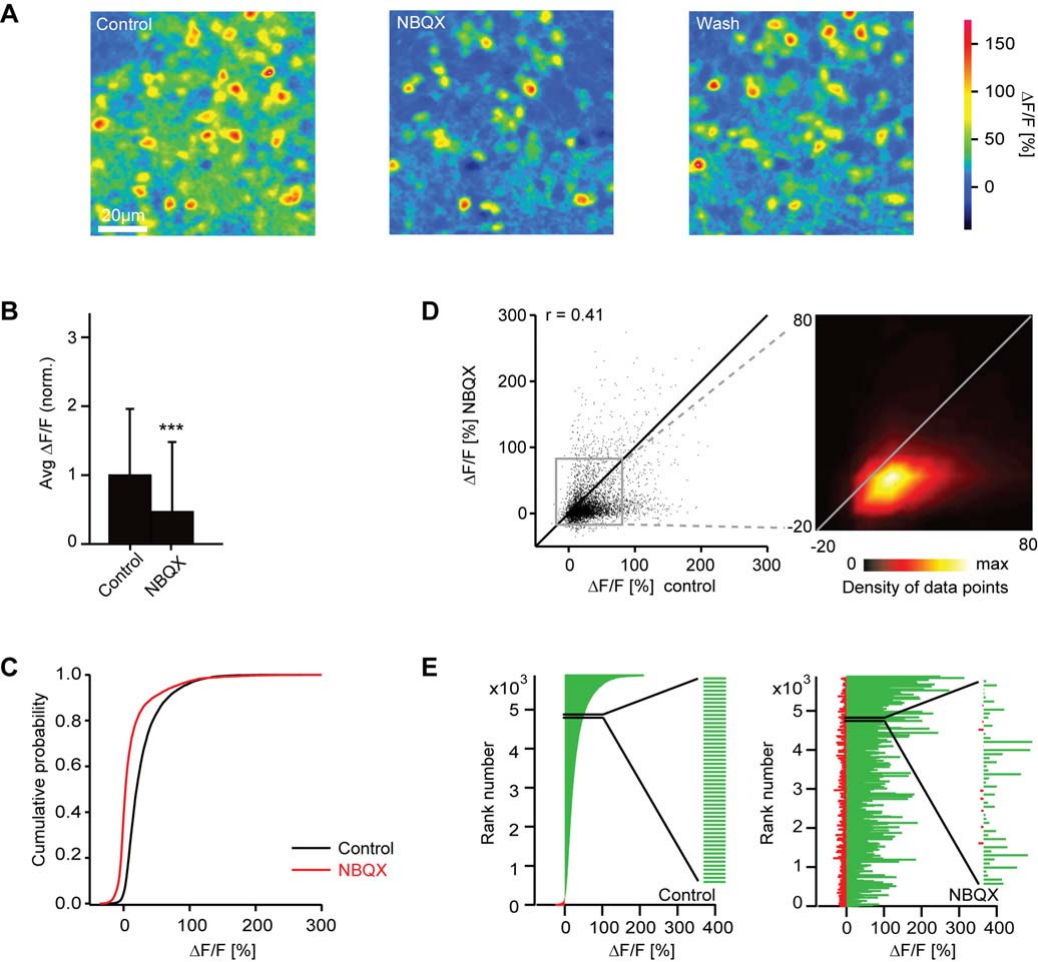
Effect of NBQX on interneuron responses measured by 2-photon Ca^2+^ imaging. (A) Odor-evoked Ca^2+^ signals in interneurons before, during and after application of NBQX (stimulus: food odor). (B) Average somatic Ca^2+^ signals before (control) and during application of NBQX, normalized to control. Error bars show standard deviation. ***, P<0.001 (sign test). (C) Cumulative distribution of Ca^2+^ signal amplitudes before (black) and during (red) application of NBQX. (D) Comparison of Ca^2+^ signal amplitudes evoked by the same odors in the same interneurons before and during application of NBQX. Data were pooled over all cells, odors and anminals (n = 5878 responses). r, Pearson correlation coefficient. Inset shows the density of data points in the boxed region. Lines are diagonals with slope one. (E) Left: interneuron odor responses ranked according to the Ca^2+^ signal before application of NBQX. Right: Responses of the same interneurons to the same odors in the presence of NBQX, ranked in the same order as in the control. Inset shows an enlargement of a subregion to demonstrate that low-amplitude values are interspersed between high amplitude values. The visual impression in the full diagram that many amplitudes are increased during NBQX treatment is therefore an artifact caused by crowding of bars in the graph.

Somata of interneurons in the deeper layers of the olfactory bulb are densely packed and show pronounced Ca^2+^ signals in response to odor stimulation (Fig. 10A) [34], [35]. In the presence of NBQX, response amplitudes of many interneurons were decreased and response patterns appeared sparser. Ca^2+^ signals in the neuropil were also substantially smaller (Fig. 10A). The average somatic Ca^2+^ signal of interneurons was significantly reduced to 47% of control (sign test: P<0.001; Fig. 10B) and the cumulative distribution of response amplitudes was shifted towards lower amplitudes (Fig. 10C). NBQX therefore increased the ratio between the mean mitral cell response and the mean interneuron response by a factor of 3.2. At the level of individual interneuron somata, the effect of NBQX was diverse. Not all responses were reduced by the same amount, and some responses were even enhanced (Fig. 10A, D, E). The correlation between activity patterns before and during NBQX treatment was 0.41 (n = 5878 responses; pooled over all interneurons and odors) and significantly lower than the correlation between activity patterns in control experiments without drugs (r = 0.71; n = 208 responses; P<0.001). Hence, blockade of AMPA/kainate receptors decreased the mean response of interneurons and changed the activity pattern across the interneuron population.

**Figure 11:**
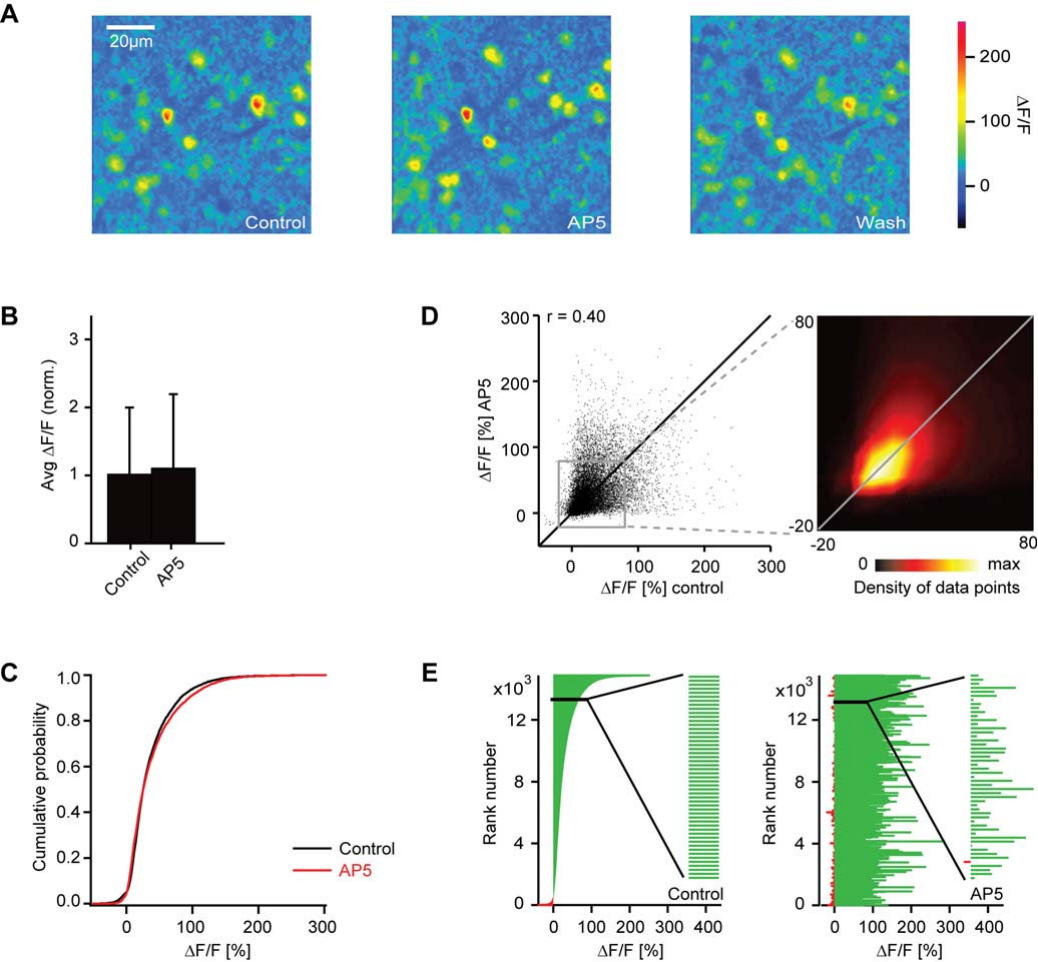
Effect of AP5 on interneuron responses measured by 2-photon Ca^2+^ imaging. (A) Odor-evoked Ca^2+^ signals in interneurons before, during and after application of AP5 (stimulus: food odor). (B) Average somatic Ca^2+^ signals before (control) and during application of AP5, normalized to control. Error bars show standard deviation. (C) Cumulative distribution of Ca^2+^ signal amplitudes before (black) and during (red) application of AP5. (D) Comparison of Ca^2+^ signal amplitudes evoked by the same odors in the same interneurons before and during application of AP5. r, Pearson correlation coefficient. Inset shows the density of data points in the boxed region. Lines are diagonals with slope one. (E) Left: interneuron odor responses ranked according to the Ca^2+^ signal before application of AP5. Data were pooled over all cells, odors and anminals (n = 14884 responses). Right: Responses of the same interneurons to the same odors in the presence of AP5, ranked in the same order as in the control. Inset shows an enlargement of a subregion to demonstrate that low-amplitude values are interspersed between high amplitude values. The visual impression in the full diagram that many amplitudes are increased during AP5 treatment is therefore an artifact caused by crowding of bars in the graph.

Blockade of NMDA receptors by AP5 caused only a slight change in the mean response amplitude of interneurons to 109% of control (sign test: P<0.001; Fig. 11A, B) and the cumulative histogram of response amplitudes remained similar (Fig. 11C). AP5 therefore changed the ratio between the mean mitral cell response and the mean interneuron response by a factor of 0.79. Individual interneuron responses, however, were often increased or decreased by AP5 (Fig. 11D, E). The correlation between activity patterns before and during AP5 treatment was 0.40 (n = 14884 responses) and significantly different from control (r = 0.71; n = 208 responses; P<0.001). Hence, the blockade of NMDA receptors had little effect on the overall amplitude of interneuron responses but caused a redistribution of activity across the population.

## Discussion

### Function of ionotropic glutamate receptors in the intact olfactory bulb

We used pharmacological manipulations in combination with electrophysiology and 2-photon Ca^2+^ imaging to study the functions of ionotropic glutamate receptors in neuronal circuits of the intact olfactory bulb. Electrophysiology records action potentials and subthreshold synaptic input at high temporal resolution while 2-photon Ca^2+^ imaging detects responses of many individual neurons simultaneously. The two recording methods therefore have complementary advantages and yielded consistent results.

The combined blockade of AMPA/kainate and NMDA receptors completely inhibited spontaneous and odor-evoked activity of mitral cells, indicating that ionotropic glutamate receptors mediate excitatory synaptic transmission from olfactory sensory neurons to mitral cells, as reported in other vertebrates [30], [31]. Paradoxically, however, the selective blockade of AMPA/kainate or NMDA receptors did not decrease the mean response of mitral cells. Rather, the AMPA/kainate receptor antagonist even increased the mean mitral cell response, and both antagonists had complex effects on neuronal responses including changes in the sign. Moreover, ionotropic glutamate receptor antagonists, particularly AP5, changed the spatio-temporal patterns of activity across mitral cell and interneuron populations. These effects cannot be explained by a partial blockade of the excitatory olfactory sensory neuron→mitral cell synapse alone but imply that ionotropic glutamate receptors influence mitral cell firing also via additional, multisynaptic pathways. The most obvious pathway is the mitral cell→interneuron→mitral cell pathway. In addition, ionotropic glutamate receptor antagonists may modulate other sub-circuits in the olfactory bulb or the feedback from higher brain regions onto interneurons (Fig. 1). Our results therefore indicate that olfactory bulb output activity is strongly influenced by synaptic pathways within and possibly beyond the olfactory bulb and provide insights into the underlying mechanisms.

### Regulation of excitation and inhibition in the olfactory bulb

Ionotropic glutamate receptors not only mediate excitatory input from olfactory sensory neurons to mitral cells but are also involved in synaptic pathways that activate interneurons, which in turn provide inhibitory input to mitral cells (Fig. 1). In the presence of an ionotropic glutamate receptor antagonist, the mean mitral cell response amplitude may therefore decrease, stay unchanged, or even increase, depending on the relative contribution of each ionotropic glutamate receptor type to each of these pathways. NBQX increased the mean response of mitral cells even though it decreases excitatory synaptic input from olfactory sensory neurons, indicating that the inhibition of AMPA/kainate receptors attenuates input from inhibitory interneurons more strongly than the excitatory input from olfactory sensory neurons. Consistent with this conclusion, NBQX significantly decreased the mean response of interneurons. The ratio between excitation and inihibition of mitral cells therefore depends on AMPA/kainate receptor-containing circuits such as the mitral cell→interneuron→mitral cell circuit or glutamatergic connections to interneurons from higher brain areas. Hence, AMPA/kainate receptor-containing circuits regulate mitral cell population activity during an odor response.

The regulation of mitral cell activity by inhibitory feedback circuits could maintain a stable level of mitral cell population activity when the strength of afferent glomerular input changes. Indeed, the total mitral cell activity converges towards a common level within a few hundred milliseconds in response to odors that evoke different amounts of activity across olfactory sensory neurons [3]. In a natural environment, the total sensory input can vary over a wide range because the number and intensity of activated glomeruli differs substantially across odors and concentrations. The AMPA/kainate receptor-containing synaptic pathways mediating inhibitory feedback onto mitral cells may therefore establish a balance between excitatory and inhibitory inputs to mitral cells that keeps the total amount of activity in the olfactory bulb within an adequate range.

The occurrence of prominent odor responses in the presence of NBQX confirms that NMDA receptors contribute to basal synaptic transmission in the olfactory bulb [36]–[38] under natural conditions. Unlike the blockade of AMPA/kainate receptors, however, the blockade of NMDA receptors had little or no effect on the mean activity of mitral cells and interneurons, indicating that NMDA receptors are less important for the regulation of mitral cell population activity than AMPA/kainate receptors. Nevertheless, the blockade of NMDA receptors changed the magnitude and time course of individual mitral cell responses. The NMDA receptors are therefore involved in shaping spatio-temporal activity patters of olfactory bulb output activity.

### AMPA/kainate receptor-dependent control of interneuron activity and mitral cell inhibition

In mammalian brain slices, NMDA receptors are critically involved in the activation of granule cells and in the recurrent inhibition of mitral cells by asynchronous GABA release from interneuron dendrites [15], [16], [19]. However, when synaptic background activity is introduced into an olfactory bulb slice, activation of granule cell firing becomes NMDA receptor-independent and asynchronous GABA release appears to be strongly diminished [39]. Under these conditions, recurrent inhibition is likely to be weak [39]. It is therefore unclear how interneuron firing and inhibition of mitral cells is controlled during an odor response in the intact olfactory bulb. We found that the mean odor-evoked somatic Ca^2+^ signal in interneurons was reduced by the blockade of AMPA/kainate receptors, but not by the blockade of NMDA receptors. Somatic Ca^2+^ signals in zebrafish mitral cells and interneurons reflect mainly action potential firing [34], [35]. Our data therefore indicate that interneuron firing is controlled primarily by AMPA/kainate receptors during an odor response.

Asynchronous GABA release from interneuron dendrites can also be triggered without action potentials, for example by Ca^2+^ influx through NMDA receptors at reciprocal synapses [15], [16], [18], [20]. It is therefore possible that NMDA receptors influence recurrent inhibition of mitral cells even if they do not strongly affect interneuron firing. If so, the blockade of NMDA receptors should increase the ratio between the mean activity of mitral cells and interneurons. However, this was not observed. Our data therefore suggest that the effect of recurrent inhibition by NMDA receptor-dependent asynchronous GABA release on mitral cell firing is, on average, weak compared to the effect of other synaptic pathways. Nevertheless, the blockade of NMDA receptors often changed individual responses of interneurons and mitral cells. Hence, NMDA receptors appear to influence odor responses in a subset of neurons and thereby cause complex effects on spatio-temporal activity patterns within the network.

Unlike inhibition of NMDA receptors, the blockade of AMPA/kainate receptors increased the mean response of mitral cells and decreased the mean somatic Ca^2+^ response of interneurons. The most likely explanation for these effects is that inhibitory input to mitral cells during an odor response is mediated primarily by AMPA/kainate receptor-dependent action potential firing of interneurons, consistent with predictions based on data from mammalian brain slices in the presence of synaptic background activity [39]. Action potentials invade large portions of the dendritic tree in interneurons [21]–[23] and are thought to trigger GABA release onto multiple postsynaptic mitral cells. Hence, lateral inhibition, rather than recurrent inhibition, may be the dominant mode of mitral cell inhibition during an odor response. Further experiments are required to test this hypothesis and to identify the synaptic pathways underlying the AMPA/kainate receptor-dependent activation of interneuron firing during an odor response.

### Effect of glutamate receptor antagonists on odor-evoked oscillatory synchronization

Oscillatory local field potential activity was abolished or reduced by the blockade of AMPA/kainate receptors or NMDA receptors, respectively, indicating that ionotropic glutamate receptors are required for the rhythmic synchronization of neuronal ensembles in the olfactory bulb. Oscillatory synchronization is thought to be mediated by networks of mitral cells and interneurons coupled by fast excitatory mitral cell→interneuron synapses and inhibitory interneuron→mitral cell synapses. Experiments in mammalian brain slices indicate that the fast inhibitory connections are mediated by GABA_A_ receptors [40]–[42] and that the fast excitatory connection is mediated by AMPA/kainate receptors [39], but the involvement of these connections in odor-evoked oscillatory synchronization has, to our knowledge, not been tested directly in the intact olfactory bulb. Our finding that AMPA/kainate receptor antagonists abolish odor-evoked oscillatory synchronization strongly supports the notion that the fast excitatory transmission in this circuit is mediated by AMPA/kainate receptors.

Because of their slow kinetics, NMDA receptors are unlikely to be directly involved in fast oscillatory synchronization in the olfactory bulb. Nevertheless, NMDA receptor antagonists reduced odor-evoked local field potential oscillations and slightly increased the oscillation frequency. One possible explanation is that NMDA receptor-mediated depolarization facilitates action potential firing of interneurons, which could enhance synchronization. However, NMDA receptor antagonists did not, on average, reduce odor-evoked Ca^2+^ signals in interneuron somata. Further experiments are therefore necessary to clarify the role of NMDA receptors in oscillatory synchronization.

### Functional implications of ionotropic glutamate receptor-containing circuits in the olfactory bulb

Synaptic pathways containing ionotropic glutamate receptors are likely to mediate important computational functions in the olfactory bulb. Under natural conditions, the number and intensity of activated glomeruli varies greatly among different odors and concentrations. The regulation of mitral cell population activity by feedback circuits involving AMPA/kainate receptors may therefore contribute to the robustness of odor representations against changes in stimulus intensity.

The dynamic reorganization of odor-evoked activity patterns during the first few hundred milliseconds of an odor response reduces the redundancy of activity patterns evoked by chemically related stimuli [1]–[3]. This decorrelation may promote odor discrimination [43] and prepare odor representations for further processing by auto-associative networks [44]. Recent results indicate that pattern decorrelation is caused, at least in part, by the “local sparsening” of mitral cell activity patterns in regions where glomerular input is dense and overlapping [35]. The most likely mechanism underlying this “local sparsening” is the spatially restricted inhibitory feedback from interneurons. Our pharmacological results are consistent with this hypothesis and indicate that ionotropic glutamate receptors in the mitral cell→interneuron→mitral cell and possibly other synaptic pathways participate in this process.

The oscillatory synchronization of odor-specific neuronal ensembles has been implicated in odor discrimination in insects [45] and affords the simultaneous transmission of different information from the olfactory bulb to higher brain regions in zebrafish [1]. Hence, the synchronization of neuronal ensembles by ionotropic glutamate receptor-containing circuits may contribute to the formatting of odor representations for read-out in higher brain regions. Together, our pharmacological analyses indicate that ionotropic glutamate receptors perform multiple functions during pattern processing in the olfactory bulb.

## Materials and Methods

### Animals, preparation, pharmacological agents and odor stimulation

Zebrafish (Danio rerio) were kept at 26.5°C at a day/night rhythm of 13/11 hours. Experiments were performed in an explant of the intact brain and nose as described previously [2], [3], [25]. Briefly, adult zebrafish (>3 months old) were cold-anaesthetized, decapitated, and olfactory forebrain structures were exposed ventrally after removal of the eyes, jaws and palate. To optimize access of drugs, the dura mater over the ventro-lateral telencephalon close to the olfactory bulb was removed with fine forceps. Care was taken to avoid damage to the olfactory bulb. The preparation was then placed in a custom made flow-chamber, continuously superfused with teleost artificial cerebro-spinal fluid [46], and warmed up to room temperature (∼22°C). All animal procedures were performed in accordance with the animal care guidelines issued by the Federal Republic of Germany.

Stock solutions of AP5 (10 mM in artificial cerebrospinal fluid) and NBQX (1 mM in DMSO; both from Tocris Bioscience, Bristol, UK) were kept frozen and diluted 1∶100–1∶200 in artificial cerebrospinal fluid immediately before the experiment, yielding final concentrations of 5–10 µM NBQX and 50–100 µM AP5. Solutions were applied through the bath. In pilot experiments, effects of different drug concentrations between 50–500 µM AP5 and 5–50 µM NBQX were compared but no qualitative differences observed, indicating that drugs penetrated well into the tissue and produced maximal effects at the concentration used in our experiments.

Odors were applied to the nasal epithelium through a constant perfusion stream using a computer-controlled, pneumatically actuated HPLC injection valve (Rheodyne, Rohnert Park, CA) as described previously [2], [25]. The volume of the applied solution and the flow rate were adjusted to obtain a stimulus duration of ∼2.4 s. Stock solutions of amino acids (Fluka, Neu-Ulm, Germany) were made in distilled water at a concentration of 1 mM, stored at −18°C, and diluted in fresh artificial cerebrospinal fluid to a final concentration of 10 µM immediately before the experiment. Extracts of commercially available dry fish food were prepared as described [25] and kept at −6°C for up to two weeks. Two hundered mg of dry food was suspended in 50 ml of artificial cerebrospinal fluid overnight, filtered through a filter paper and diluted 1∶100 in artificial cerebrospinal fluid immediately before the experiment.

### Electrophysiological recordings

Electrophysiological recordings from mitral cells were performed in the ventro-lateral olfactory bulb where amino acid-responsive neurons are located [2], [29]. Borosilicate patch pipettes (8–13 MΩ) were pulled on a P-2000 electrode puller (Sutter Instruments, Novato, CA) and filled with intracellular solution containing (in mM): 130 K-gluconate, 10 Na-gluconate, 10 Na-phosphocreatine, 4 NaCl, 4 Mg-ATP, 0.3 Na-GTP, 10 HEPES (pH 7.25; ∼300 mosm). Cells in the olfactory bulb were visualized by differential interference contrast video microscopy or similar methods through a coverslip in the bottom of the chamber. Recorded neurons were selected for their large soma diameter (∼10 µm) and a position close in the glomerular/mitral cell layer. Anatomical studies demonstrated that these are characteristics of mitral cells [32], [34], [37], [47]. Membrane potential values were corrected for a junction potential of −13 mV.

Recordings were performed using an Axoclamp 2B amplifier (Axon Instruments) and digitized at 10 kHz using National Instruments hardware and custom software written in IgorPro (Wavemetrics). To prevent early clogging of the glass capillary tip, a pressure of ∼100 mbar was applied to the pipette interior during penetration of the tissue and lowered to ∼40 mbar before a target cell was approached. After formation of a Giga-seal and break-in, intracellular whole-cell recordings were performed in current clamp mode (n = 16 mitral cells). In most cells, a small negative holding current was applied to stabilize recordings.

When Giga-seal formation or break-in could not be achieved, pipette pressure was released and extracellular recordings were performed in the loose-patch or cell-attached mode (n = 14 mitral cells). Two whole-cell recordings were lost during the experiment and continued as loose-patch recordings. Spontaneous firing rates of cells recorded in whole-cell mode (3.9±3.6 Hz; mean±standard deviation) were slightly lower than those recorded extracellularly (7.3±6.0 Hz), probably due to the holding current. When a recording was established, typically 2 food extracts and 6 amino acids were applied to select one or two stimuli that evoked a strong response in the recorded cell. These two stimuli were then applied 3–8 times (usually five times) at 2 min intervals in a pseudo-randomly interleaved sequence, followed by ∼15 min without odor stimulation to wash in drugs. The original stimulus sequence was then repeated. Completion of this stimulus protocol required continuous recordings for approximately 60 min. Recordings that were not stable up to this point, as judged by the measured resting potential and action potential amplitude, were excluded from the analysis. Drugs were then washed out for at least 30 min. When recordings were still stable, the stimulus sequence was repeated again. In total, responses of 30 neurons to 54 odor stimuli were measured before and during drug application, and 21 of these responses were tested again after wash-out.

To measure odor-evoked oscillatory activity in the local field potential, glass micropipettes were filled with artificial cerebrospinal fluid (8–13 MΩ) and positioned in the glomerular/mitral cell layer. Recordings were made in bridge mode using an Axoclamp 2B amplifier (Axon Instruments) and band-pass filtered offline between 8–43 Hz. The position of the micropipette was optimized by small movements of the capillary tip while measuring oscillation amplitudes during stimulation with food extract.

### Two-photon Ca^2+^ **imaging**

Two-photon Ca^2+^ imaging experiments were performed in transgenic fish expressing yellow cameleon (YC) under the control of a fragment of the HuC promoter (HuC-YC) [33]. In the adult olfactory bulb, HuC-YC is expressed selectively in mitral cells [32]. HuC-YC-negative cells were collectively classified as interneurons and include periglomerular and granule cells. YC fluorescence did not change in response to odor stimulation and was exclusively used as an anatomical marker.

The red-fluorescent Ca^2+^ indicator, rhod-2-AM ester (Invitrogen/Molecular Probes), was injected into the olfactory bulb as described previously [34]. Briefly, 50 µg of rhod-2-AM was dissolved in 16 µl DMSO/pluronic acid (80/20) and this solution was diluted 1∶10 in artificial cerebrospinal fluid before the experiment. The dye solution was loaded into a patch pipette after gently breaking off the very tip and pressure-injected into the olfactory bulb under fluorescence optics. Injections were terminated when a predetermined fluorescence intensity level was reached to avoid excessive dye loading. To label mitral cells, multiple brief injections were made into the glomerular/mitral cell layer at different sites. To label interneurons, injections were made into the granule cell layer. For details see [34].

Fluorescence images were acquired using a custom-built 2-photon microscope [48] equipped with a 20× water immersion objective (NA 0.95; Olympus). Two-photon fluorescence was excited at 830 nm by a mode-locked Ti:Sapphire laser (Mira900; 76 MHz; Coherent, Santa Clara, CA) pumped by a 10 W diode laser (Verdi; Coherent). Fluorescence emission was detected externally by a photomultiplier-based whole-field detector in two wavelength channels (515/30 nm and 610/75 nm), allowing for the separate and simultaneous detection of HuC-YC and rhod-2 fluorescence, respectively. Image acquisition was controlled by custom software (CFNT; written by Ray Stepnoski at Bell Labs, Murry Hill, NJ, and Michael Müller at the Max-Planck-Institute for Medical Research, Heidelberg, Germany). Laser intensity was adjusted to minimize photobleaching.

To measure Ca^2+^ signals, series of images from a single focal plane were acquired at 128 ms/frame and 128×256 pixels or 256 ms/frame and 256×256 pixels. Previous experiments demonstrated that odor-evoked patterns of Ca^2+^ signals measured with this protocol are reproducible and stable over hours [35]. To verify the stability of responses, we repeated the first stimulus in the sequence at least once before the drug treatment was started, and typically multiple times during the initial phase of the experiment. When responses appeared not stable, experiments were discarded. Responses of interneurons were recorded in deep layers of the olfactory bulb that contain predominantly granule cells. The focal plane was kept constant during an experiment. Slow drift was corrected if necessary using natural landmarks in the raw fluorescence image and in the HuC-YC fluorescence image.

Image series of raw rhod-2 fluorescence were converted into image series representing the fractional change in pixel intensity relative to a pre-stimulus baseline (ΔF/F). Response maps were constructed by averaging ΔF/F images during a period of 5s around response peak and mild spatial low-pass filtering using a Gaussian kernel (width, 5 pixels; σ, 1.2 pixels).

### Data analysis

Data were analyzed off-line using routines written in IgorPro (Wavemetrics) or Matlab (Mathworks). Trains of action potentials were described as series of delta functions and convolved with a Gaussian kernel (σ = 200 ms; other values gave similar results) to obtain firing rate functions. Firing rate functions from repeated stimulus applications were averaged, yielding peri-stimulus time histograms. Spontaneous firing rates were measured during two seconds before stimulus onset and averaged over all trials measured at a given condition (usually ≥10 trials). Effects of drugs on odor responses were assessed by subtracting peri-stimulus time histograms measured in the presence of a drug from corresponding peri-stimulus time histograms before drug application.

Mean response amplitudes of single neurons measured under different conditions were compared using a paired Student's t-test because measurements in repeated trials were approximally normally distributed. Mean responses across populations of neurons were usually not normally distributed and compared using a non-parametric sign test. To test for statistical differences between correlation strengths, correlation coefficients were transformed using the Fisher Z transform and compared using the z statistic.
